# Correction: Queen cells acceptance rate and royal jelly production in worker honey bees of two Apis mellifera races

**DOI:** 10.1371/journal.pone.0270445

**Published:** 2022-06-21

**Authors:** Khalid Ali Khan, Hamed A. Ghramh, Zubair Ahmad, Mogbel A. A. El-Niweiri, Mohamed Elimam Ahamed Mohammed

The affiliations for the 2nd author are not indicated. Hamed A. Ghramh is affiliated with: Research Center for Advanced Materials Science (RCAMS), King Khalid University, Abha, Saudi Arabia; Unit of Bee Research and Honey Production, Faculty of Science, King Khalid University, Abha, Saudi Arabia; and Biology Department, Faculty of Science, King Khalid University, Abha, Saudi Arabia.
